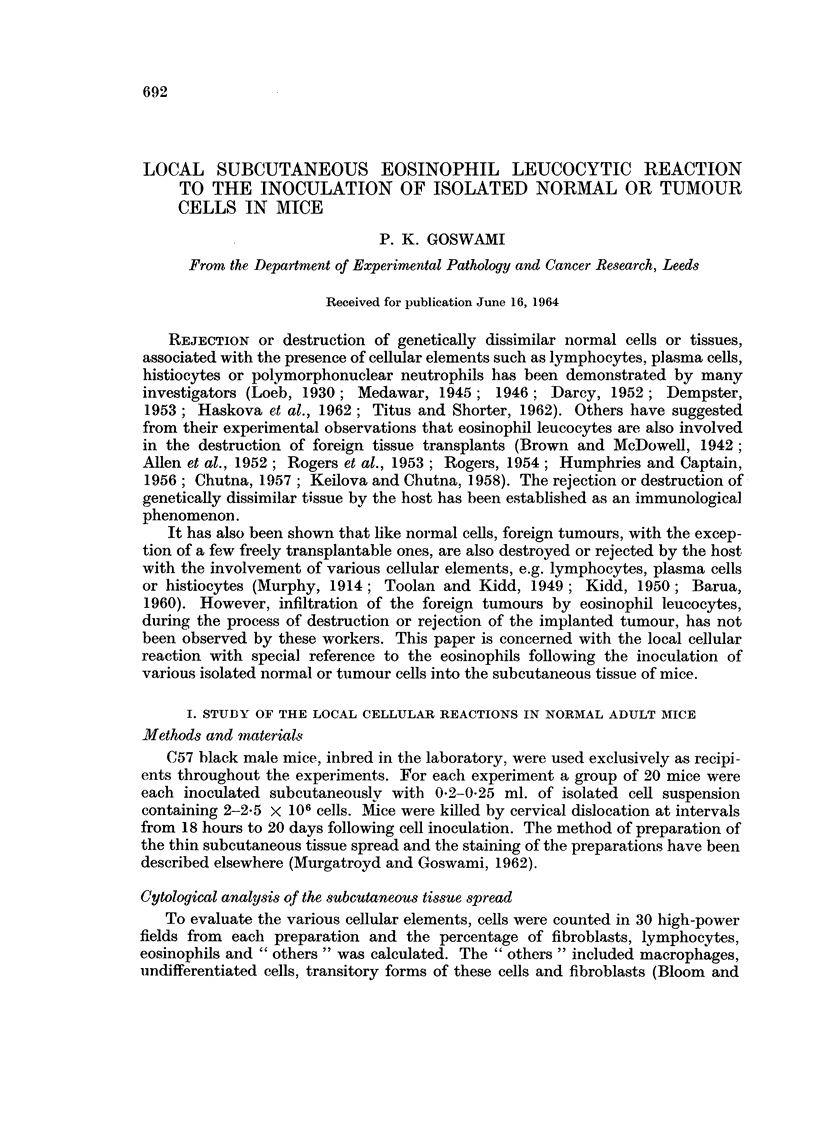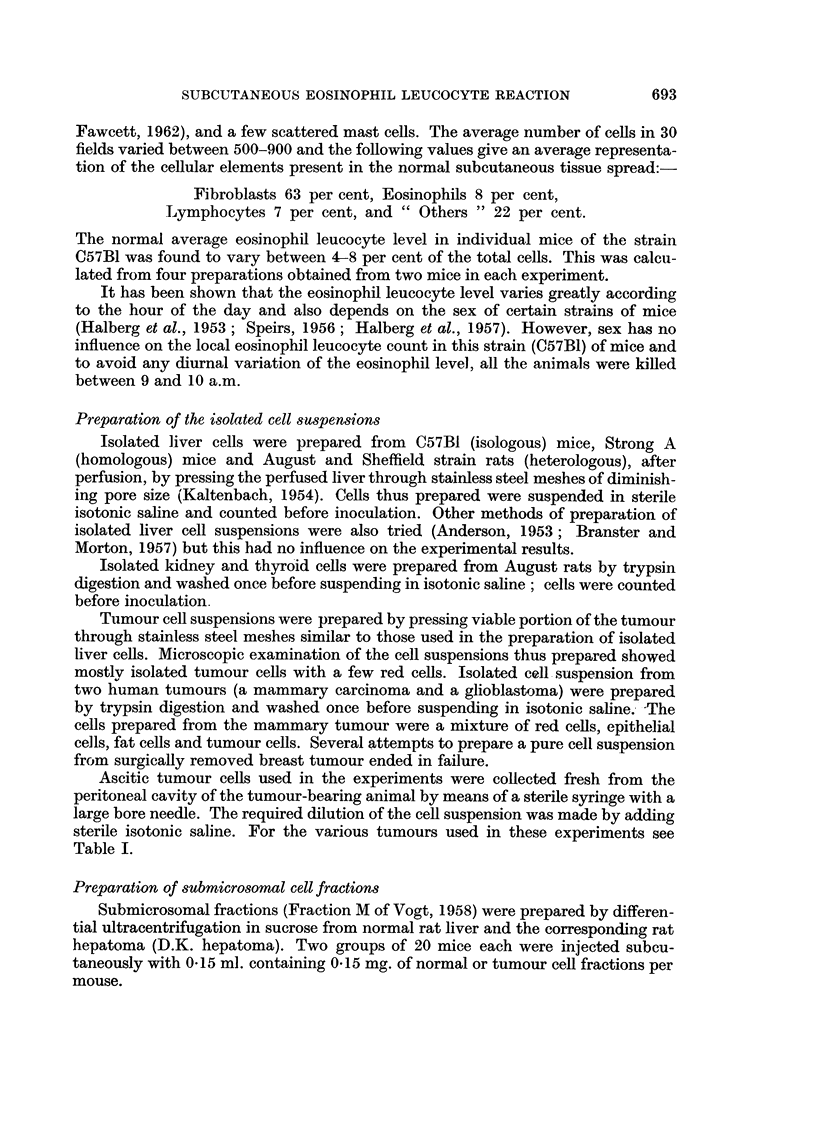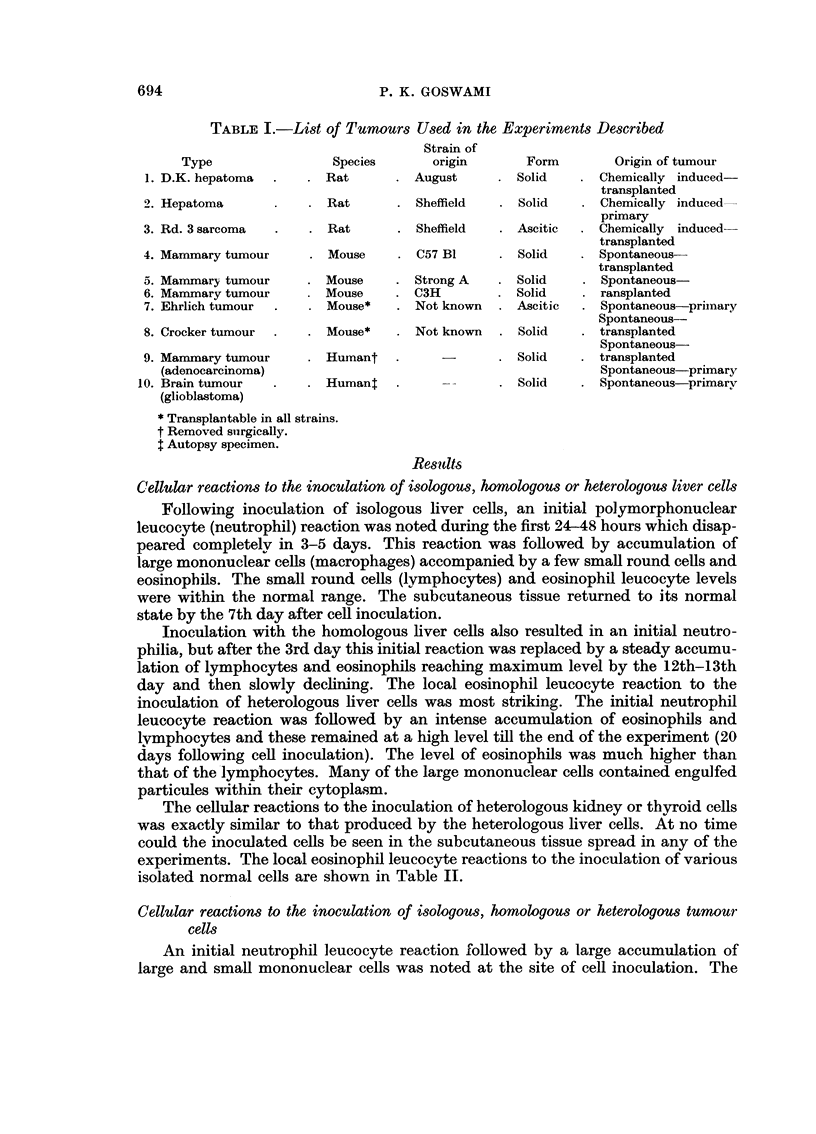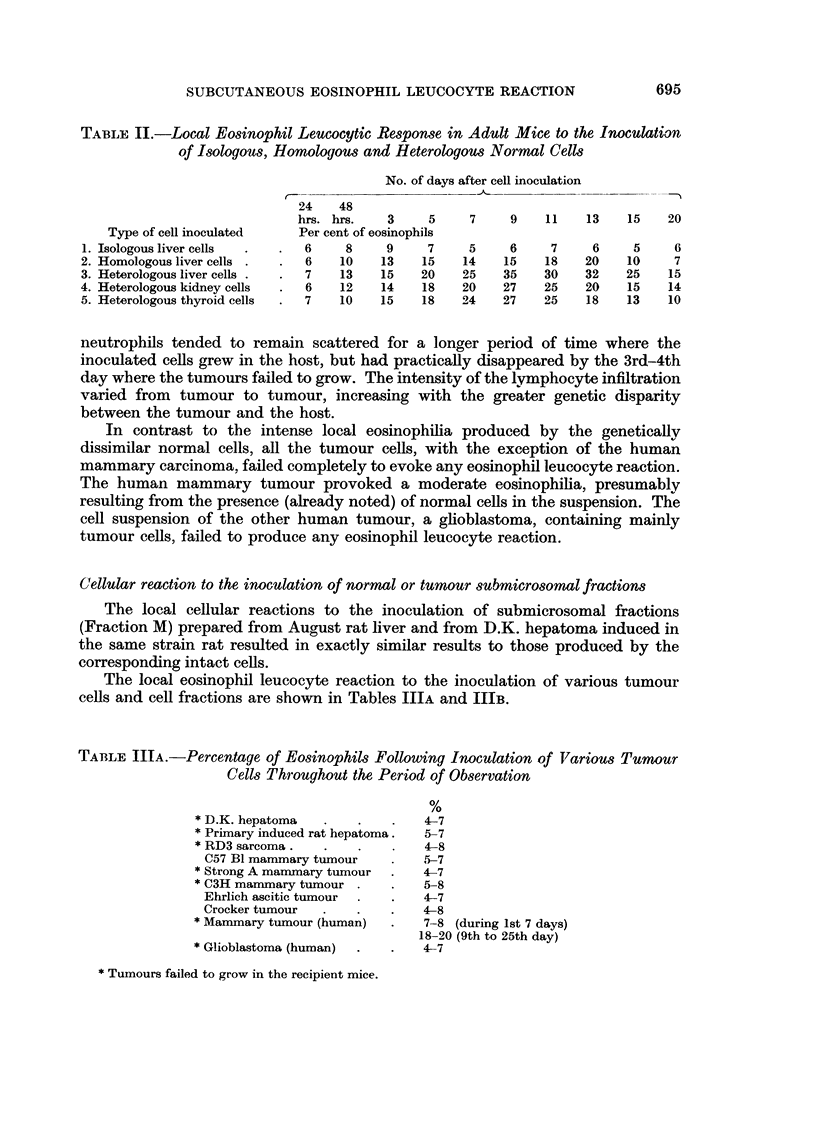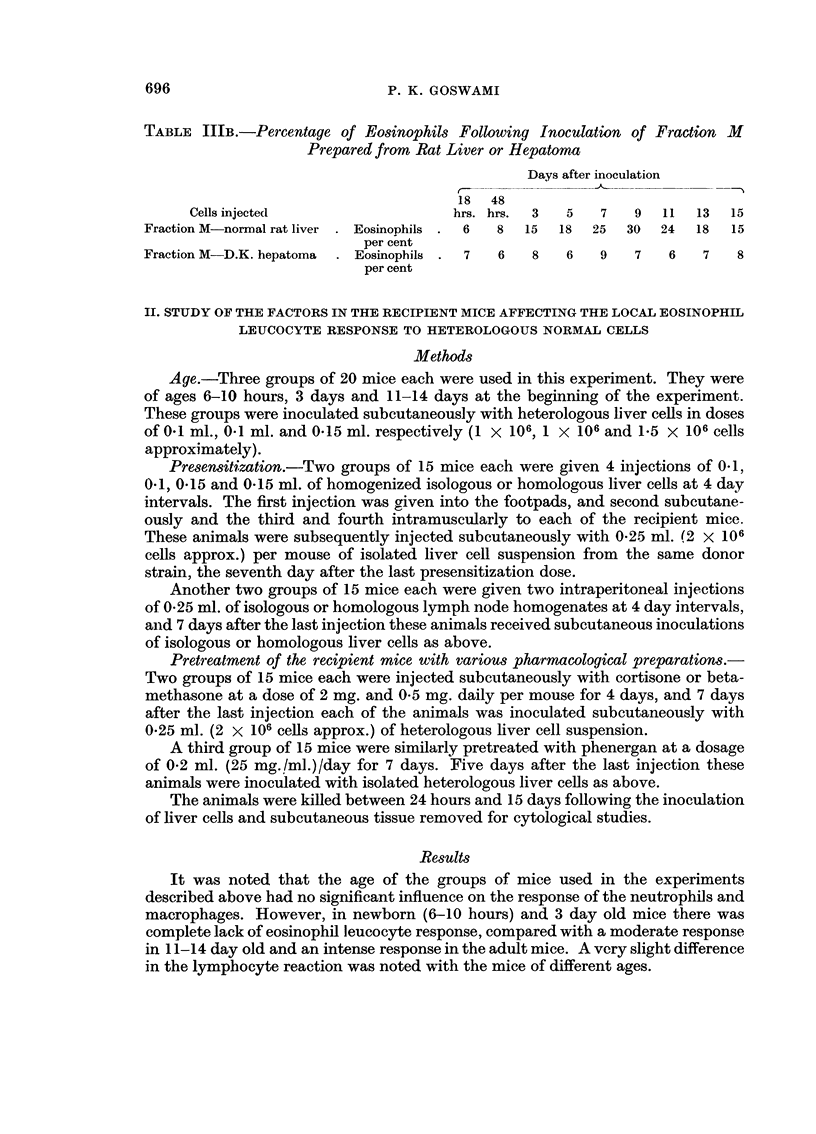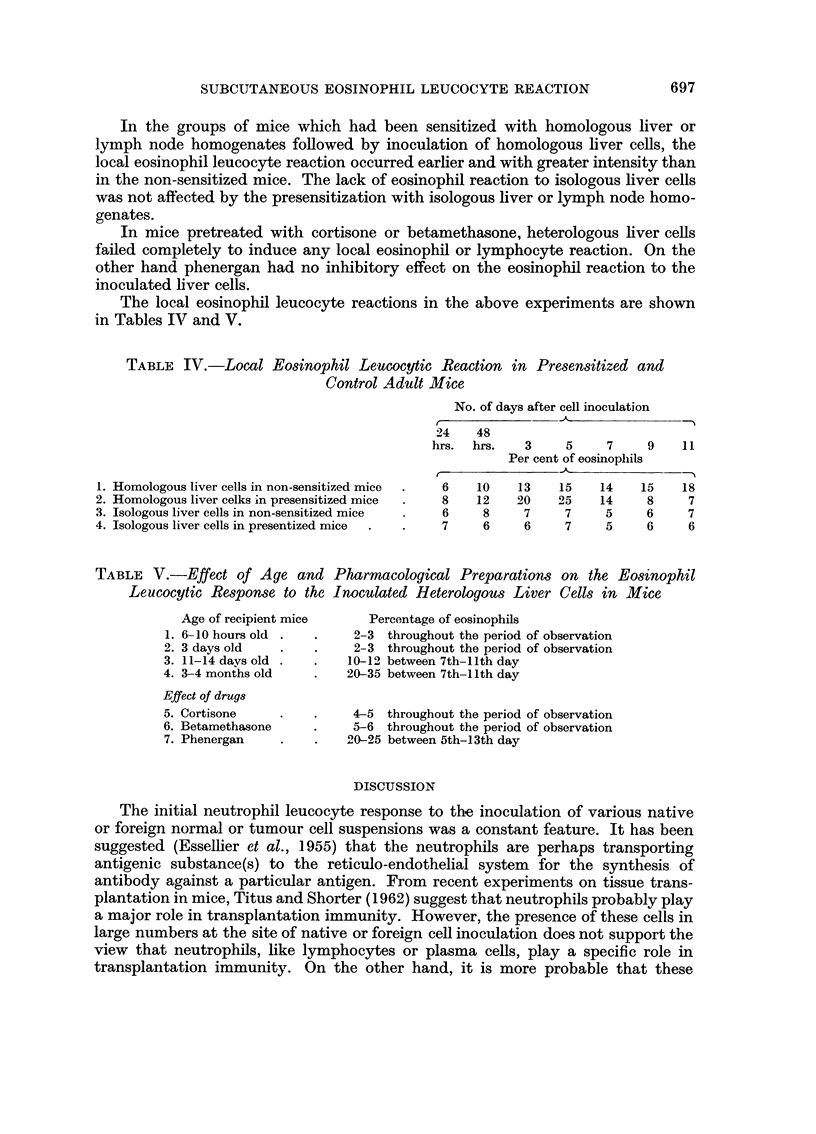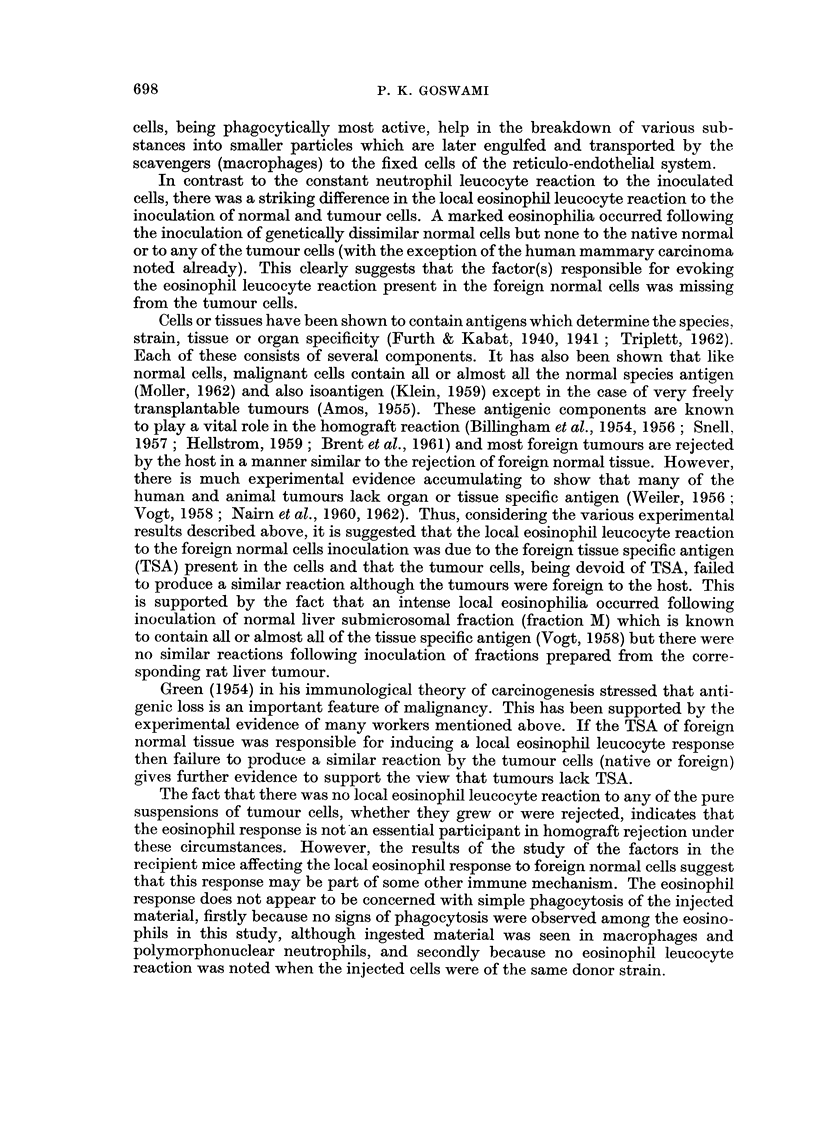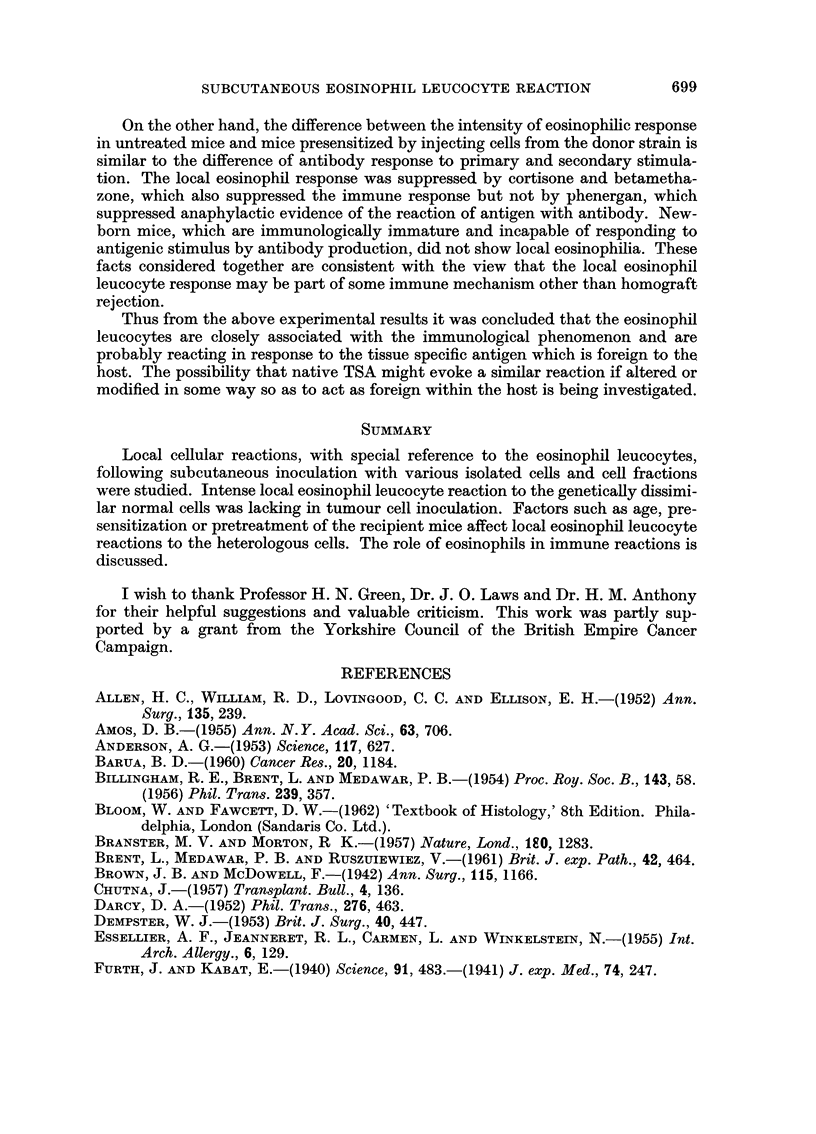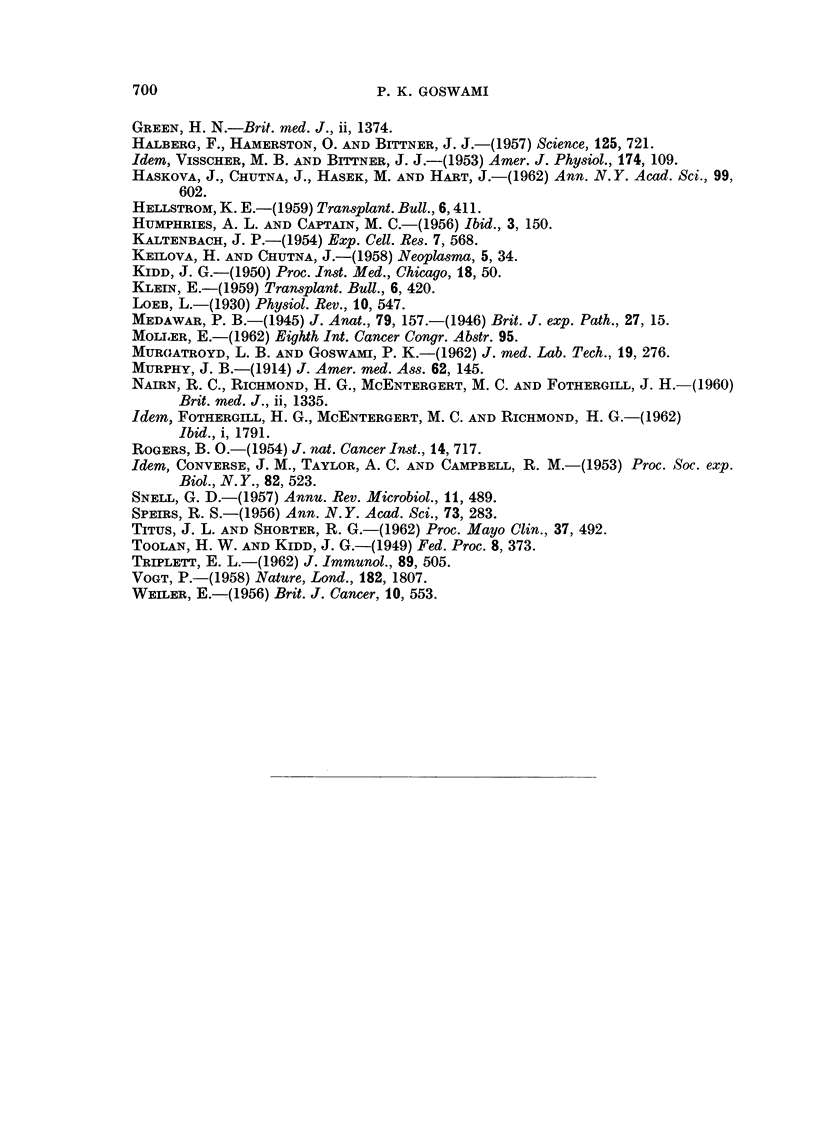# Local Subcutaneous Eosinophil Leucocytic Reaction to the Inoculation of Isolated Normal or Tumour Cells in Mice

**DOI:** 10.1038/bjc.1964.80

**Published:** 1964-12

**Authors:** P. K. Goswami


### LOCAL SUBCUTANEOUS EOSINOPHIL LEUCOCYTIC REACTION TO THE INOCULATION OF ISOLATED NORMAL OR TUMOUR CELLS IN MICE

P. K. GOSWAMI

*From the Department of Experimental Pathology and Cancer Research, Leeds*

Received for publication June 16, 1964

REJECTION or destruction of genetically dissimilar normal cells or tissues, associated with the presence of cellular elements such as lymphocytes, plasma cells, histiocytes or polymorphonuclear neutrophils has been demonstrated by many investigators (Loeb, 1930; Medawar, 1945; 1946; Darcy, 1952; Dempster, 1953; Haskova *et al.*, 1962; Titus and Shorter, 1962). Others have suggested from their experimental observations that eosinophil leucocytes are also involved in the destruction of foreign tissue transplants (Brown and McDowell, 1942; Allen *et al.*, 1952; Rogers *et al.*, 1953; Rogers, 1954; Humphries and Captain, 1956; Chutna, 1957; Keilova and Chutna, 1958). The rejection or destruction of genetically dissimilar tissue by the host has been established as an immunological phenomenon.

It has also been shown that like normal cells, foreign tumours, with the exception of a few freely transplantable ones, are also destroyed or rejected by the host with the involvement of various cellular elements, e.g. lymphocytes, plasma cells or histiocytes (Murphy, 1914; Toolan and Kidd, 1949; Kidd, 1950; Barua, 1960). However, infiltration of the foreign tumours by eosinophil leucocytes, during the process of destruction or rejection of the implanted tumour, has not been observed by these workers. This paper is concerned with the local cellular reaction with special reference to the eosinophils following the inoculation of various isolated normal or tumour cells into the subcutaneous tissue of mice.

### I. STUDY OF THE LOCAL CELLULAR REACTIONS IN NORMAL ADULT MICE

### *Methods and materials*

C57 black male mice, inbred in the laboratory, were used exclusively as recipients throughout the experiments. For each experiment a group of 20 mice were each inoculated subcutaneously with 0·2–0·25 ml. of isolated cell suspension containing 2–2·5 $\times$ $10^6$ cells. Mice were killed by cervical dislocation at intervals from 18 hours to 20 days following cell inoculation. The method of preparation of the thin subcutaneous tissue spread and the staining of the preparations have been described elsewhere (Murgatroyd and Goswami, 1962).

### *Cytological analysis of the subcutaneous tissue spread*

To evaluate the various cellular elements, cells were counted in 30 high-power fields from each preparation and the percentage of fibroblasts, lymphocytes, eosinophils and " others " was calculated. The " others " included macrophages, undifferentiated cells, transitory forms of these cells and fibroblasts (Bloom and

Fawcett, 1962), and a few scattered mast cells. The average number of cells in 30 fields varied between 500–900 and the following values give an average representation of the cellular elements present in the normal subcutaneous tissue spread:—

Fibroblasts 63 per cent, Eosinophils 8 per cent,
Lymphocytes 7 per cent, and " Others " 22 per cent.

The normal average eosinophil leucocyte level in individual mice of the strain C57Bl was found to vary between 4–8 per cent of the total cells. This was calculated from four preparations obtained from two mice in each experiment.

It has been shown that the eosinophil leucocyte level varies greatly according to the hour of the day and also depends on the sex of certain strains of mice (Halberg *et al.*, 1953 ; Speirs, 1956 ; Halberg *et al.*, 1957). However, sex has no influence on the local eosinophil leucocyte count in this strain (C57Bl) of mice and to avoid any diurnal variation of the eosinophil level, all the animals were killed between 9 and 10 a.m.

*Preparation of the isolated cell suspensions*

Isolated liver cells were prepared from C57Bl (isologous) mice, Strong A (homologous) mice and August and Sheffield strain rats (heterologous), after perfusion, by pressing the perfused liver through stainless steel meshes of diminishing pore size (Kaltenbach, 1954). Cells thus prepared were suspended in sterile isotonic saline and counted before inoculation. Other methods of preparation of isolated liver cell suspensions were also tried (Anderson, 1953 ; Branster and Morton, 1957) but this had no influence on the experimental results.

Isolated kidney and thyroid cells were prepared from August rats by trypsin digestion and washed once before suspending in isotonic saline ; cells were counted before inoculation.

Tumour cell suspensions were prepared by pressing viable portion of the tumour through stainless steel meshes similar to those used in the preparation of isolated liver cells. Microscopic examination of the cell suspensions thus prepared showed mostly isolated tumour cells with a few red cells. Isolated cell suspension from two human tumours (a mammary carcinoma and a glioblastoma) were prepared by trypsin digestion and washed once before suspending in isotonic saline. The cells prepared from the mammary tumour were a mixture of red cells, epithelial cells, fat cells and tumour cells. Several attempts to prepare a pure cell suspension from surgically removed breast tumour ended in failure.

Ascitic tumour cells used in the experiments were collected fresh from the peritoneal cavity of the tumour-bearing animal by means of a sterile syringe with a large bore needle. The required dilution of the cell suspension was made by adding sterile isotonic saline. For the various tumours used in these experiments see Table I.

*Preparation of submicrosomal cell fractions*

Submicrosomal fractions (Fraction M of Vogt, 1958) were prepared by differential ultracentrifugation in sucrose from normal rat liver and the corresponding rat hepatoma (D.K. hepatoma). Two groups of 20 mice each were injected subcutaneously with 0·15 ml. containing 0·15 mg. of normal or tumour cell fractions per mouse.

TABLE I.—*List of Tumours Used in the Experiments Described*

| Type | Species | Strain of origin | Form | Origin of tumour |
|---|---|---|---|---|
| 1. D.K. hepatoma | Rat | August | Solid | Chemically induced—transplanted |
| 2. Hepatoma | Rat | Sheffield | Solid | Chemically induced—primary |
| 3. Rd. 3 sarcoma | Rat | Sheffield | Ascitic | Chemically induced—transplanted |
| 4. Mammary tumour | Mouse | C57 Bl | Solid | Spontaneous—transplanted |
| 5. Mammary tumour | Mouse | Strong A | Solid | Spontaneous— |
| 6. Mammary tumour | Mouse | C3H | Solid | ransplanted |
| 7. Ehrlich tumour | Mouse* | Not known | Ascitic | Spontaneous—primary |
| 8. Crocker tumour | Mouse* | Not known | Solid | Spontaneous—transplanted |
| 9. Mammary tumour (adenocarcinoma) | Human† | — | Solid | Spontaneous—transplanted |
| | | | | Spontaneous—primary |
| 10. Brain tumour (glioblastoma) | Human‡ | — | Solid | Spontaneous—primary |

* Transplantable in all strains.

† Removed surgically.

‡ Autopsy specimen.

### *Results*

### *Cellular reactions to the inoculation of isologous, homologous or heterologous liver cells*

Following inoculation of isologous liver cells, an initial polymorphonuclear leucocyte (neutrophil) reaction was noted during the first 24–48 hours which disappeared completely in 3–5 days. This reaction was followed by accumulation of large mononuclear cells (macrophages) accompanied by a few small round cells and eosinophils. The small round cells (lymphocytes) and eosinophil leucocyte levels were within the normal range. The subcutaneous tissue returned to its normal state by the 7th day after cell inoculation.

Inoculation with the homologous liver cells also resulted in an initial neutrophilia, but after the 3rd day this initial reaction was replaced by a steady accumulation of lymphocytes and eosinophils reaching maximum level by the 12th–13th day and then slowly declining. The local eosinophil leucocyte reaction to the inoculation of heterologous liver cells was most striking. The initial neutrophil leucocyte reaction was followed by an intense accumulation of eosinophils and lymphocytes and these remained at a high level till the end of the experiment (20 days following cell inoculation). The level of eosinophils was much higher than that of the lymphocytes. Many of the large mononuclear cells contained engulfed particules within their cytoplasm.

The cellular reactions to the inoculation of heterologous kidney or thyroid cells was exactly similar to that produced by the heterologous liver cells. At no time could the inoculated cells be seen in the subcutaneous tissue spread in any of the experiments. The local eosinophil leucocyte reactions to the inoculation of various isolated normal cells are shown in Table II.

### *Cellular reactions to the inoculation of isologous, homologous or heterologous tumour cells*

An initial neutrophil leucocyte reaction followed by a large accumulation of large and small mononuclear cells was noted at the site of cell inoculation. The

TABLE II.—*Local Eosinophil Leucocytic Response in Adult Mice to the Inoculation of Isologous, Homologous and Heterologous Normal Cells*

| | No. of days after cell inoculation | | | | | | | | | |
|---|---|---|---|---|---|---|---|---|---|---|
| | 24 hrs. | 48 hrs. | 3 | 5 | 7 | 9 | 11 | 13 | 15 | 20 |
| Type of cell inoculated | Per cent of eosinophils | | | | | | | | | |
| 1. Isologous liver cells | 6 | 8 | 9 | 7 | 5 | 6 | 7 | 6 | 5 | 6 |
| 2. Homologous liver cells | 6 | 10 | 13 | 15 | 14 | 15 | 18 | 20 | 10 | 7 |
| 3. Heterologous liver cells | 7 | 13 | 15 | 20 | 25 | 35 | 30 | 32 | 25 | 15 |
| 4. Heterologous kidney cells | 6 | 12 | 14 | 18 | 20 | 27 | 25 | 20 | 15 | 14 |
| 5. Heterologous thyroid cells | 7 | 10 | 15 | 18 | 24 | 27 | 25 | 18 | 13 | 10 |

neutrophils tended to remain scattered for a longer period of time where the inoculated cells grew in the host, but had practically disappeared by the 3rd–4th day where the tumours failed to grow. The intensity of the lymphocyte infiltration varied from tumour to tumour, increasing with the greater genetic disparity between the tumour and the host.

In contrast to the intense local eosinophilia produced by the genetically dissimilar normal cells, all the tumour cells, with the exception of the human mammary carcinoma, failed completely to evoke any eosinophil leucocyte reaction. The human mammary tumour provoked a moderate eosinophilia, presumably resulting from the presence (already noted) of normal cells in the suspension. The cell suspension of the other human tumour, a glioblastoma, containing mainly tumour cells, failed to produce any eosinophil leucocyte reaction.

*Cellular reaction to the inoculation of normal or tumour submicrosomal fractions*

The local cellular reactions to the inoculation of submicrosomal fractions (Fraction M) prepared from August rat liver and from D.K. hepatoma induced in the same strain rat resulted in exactly similar results to those produced by the corresponding intact cells.

The local eosinophil leucocyte reaction to the inoculation of various tumour cells and cell fractions are shown in Tables IIIA and IIIB.

TABLE IIIA.—*Percentage of Eosinophils Following Inoculation of Various Tumour Cells Throughout the Period of Observation*

| | % | |
|---|---|---|
| * D.K. hepatoma | 4–7 | |
| * Primary induced rat hepatoma | 5–7 | |
| * RD3 sarcoma | 4–8 | |
| C57 Bl mammary tumour | 5–7 | |
| * Strong A mammary tumour | 4–7 | |
| * C3H mammary tumour | 5–8 | |
| Ehrlich ascitic tumour | 4–7 | |
| Crocker tumour | 4–8 | |
| * Mammary tumour (human) | 7–8 | (during 1st 7 days) |
| | 18–20 | (9th to 25th day) |
| * Glioblastoma (human) | 4–7 | |

* Tumours failed to grow in the recipient mice.

TABLE IIIB.—*Percentage of Eosinophils Following Inoculation of Fraction M Prepared from Rat Liver or Hepatoma*

| Cells injected | | Days after inoculation 18 hrs. | 48 hrs. | 3 | 5 | 7 | 9 | 11 | 13 | 15 |
|---|---|---|---|---|---|---|---|---|---|---|
| Fraction M—normal rat liver | Eosinophils per cent | 6 | 8 | 15 | 18 | 25 | 30 | 24 | 18 | 15 |
| Fraction M—D.K. hepatoma | Eosinophils per cent | 7 | 6 | 8 | 6 | 9 | 7 | 6 | 7 | 8 |

### II. STUDY OF THE FACTORS IN THE RECIPIENT MICE AFFECTING THE LOCAL EOSINOPHIL LEUCOCYTE RESPONSE TO HETEROLOGOUS NORMAL CELLS

### *Methods*

*Age.*—Three groups of 20 mice each were used in this experiment. They were of ages 6–10 hours, 3 days and 11–14 days at the beginning of the experiment. These groups were inoculated subcutaneously with heterologous liver cells in doses of 0·1 ml., 0·1 ml. and 0·15 ml. respectively ($1 \times 10^6$, $1 \times 10^6$ and $1{\cdot}5 \times 10^6$ cells approximately).

*Presensitization.*—Two groups of 15 mice each were given 4 injections of 0·1, 0·1, 0·15 and 0·15 ml. of homogenized isologous or homologous liver cells at 4 day intervals. The first injection was given into the footpads, and second subcutaneously and the third and fourth intramuscularly to each of the recipient mice. These animals were subsequently injected subcutaneously with 0·25 ml. ($2 \times 10^6$ cells approx.) per mouse of isolated liver cell suspension from the same donor strain, the seventh day after the last presensitization dose.

Another two groups of 15 mice each were given two intraperitoneal injections of 0·25 ml. of isologous or homologous lymph node homogenates at 4 day intervals, and 7 days after the last injection these animals received subcutaneous inoculations of isologous or homologous liver cells as above.

*Pretreatment of the recipient mice with various pharmacological preparations.*—Two groups of 15 mice each were injected subcutaneously with cortisone or betamethasone at a dose of 2 mg. and 0·5 mg. daily per mouse for 4 days, and 7 days after the last injection each of the animals was inoculated subcutaneously with 0·25 ml. ($2 \times 10^6$ cells approx.) of heterologous liver cell suspension.

A third group of 15 mice were similarly pretreated with phenergan at a dosage of 0·2 ml. (25 mg./ml.)/day for 7 days. Five days after the last injection these animals were inoculated with isolated heterologous liver cells as above.

The animals were killed between 24 hours and 15 days following the inoculation of liver cells and subcutaneous tissue removed for cytological studies.

### *Results*

It was noted that the age of the groups of mice used in the experiments described above had no significant influence on the response of the neutrophils and macrophages. However, in newborn (6–10 hours) and 3 day old mice there was complete lack of eosinophil leucocyte response, compared with a moderate response in 11–14 day old and an intense response in the adult mice. A very slight difference in the lymphocyte reaction was noted with the mice of different ages.

In the groups of mice which had been sensitized with homologous liver or lymph node homogenates followed by inoculation of homologous liver cells, the local eosinophil leucocyte reaction occurred earlier and with greater intensity than in the non-sensitized mice. The lack of eosinophil reaction to isologous liver cells was not affected by the presensitization with isologous liver or lymph node homogenates.

In mice pretreated with cortisone or betamethasone, heterologous liver cells failed completely to induce any local eosinophil or lymphocyte reaction. On the other hand phenergan had no inhibitory effect on the eosinophil reaction to the inoculated liver cells.

The local eosinophil leucocyte reactions in the above experiments are shown in Tables IV and V.

TABLE IV.—*Local Eosinophil Leucocytic Reaction in Presensitized and Control Adult Mice*

| | No. of days after cell inoculation | | | | | | |
|---|---|---|---|---|---|---|---|
| | 24 hrs. | 48 hrs. | 3 | 5 | 7 | 9 | 11 |
| | Per cent of eosinophils | | | | | | |
| 1. Homologous liver cells in non-sensitized mice . | 6 | 10 | 13 | 15 | 14 | 15 | 18 |
| 2. Homologous liver celks in presensitized mice . | 8 | 12 | 20 | 25 | 14 | 8 | 7 |
| 3. Isologous liver cells in non-sensitized mice . | 6 | 8 | 7 | 7 | 5 | 6 | 7 |
| 4. Isologous liver cells in presentized mice . . | 7 | 6 | 6 | 7 | 5 | 6 | 6 |

TABLE V.—*Effect of Age and Pharmacological Preparations on the Eosinophil Leucocytic Response to the Inoculated Heterologous Liver Cells in Mice*

| Age of recipient mice | Percentage of eosinophils |
|---|---|
| 1. 6–10 hours old . . | 2–3 throughout the period of observation |
| 2. 3 days old . . | 2–3 throughout the period of observation |
| 3. 11–14 days old . . | 10–12 between 7th–11th day |
| 4. 3–4 months old . | 20–35 between 7th–11th day |
| *Effect of drugs* | |
| 5. Cortisone . . | 4–5 throughout the period of observation |
| 6. Betamethasone . | 5–6 throughout the period of observation |
| 7. Phenergan . . | 20–25 between 5th–13th day |

### DISCUSSION

The initial neutrophil leucocyte response to the inoculation of various native or foreign normal or tumour cell suspensions was a constant feature. It has been suggested (Essellier *et al.*, 1955) that the neutrophils are perhaps transporting antigenic substance(s) to the reticulo-endothelial system for the synthesis of antibody against a particular antigen. From recent experiments on tissue transplantation in mice, Titus and Shorter (1962) suggest that neutrophils probably play a major role in transplantation immunity. However, the presence of these cells in large numbers at the site of native or foreign cell inoculation does not support the view that neutrophils, like lymphocytes or plasma cells, play a specific role in transplantation immunity. On the other hand, it is more probable that these cells, being phagocytically most active, help in the breakdown of various substances into smaller particles which are later engulfed and transported by the scavengers (macrophages) to the fixed cells of the reticulo-endothelial system.

In contrast to the constant neutrophil leucocyte reaction to the inoculated cells, there was a striking difference in the local eosinophil leucocyte reaction to the inoculation of normal and tumour cells. A marked eosinophilia occurred following the inoculation of genetically dissimilar normal cells but none to the native normal or to any of the tumour cells (with the exception of the human mammary carcinoma noted already). This clearly suggests that the factor(s) responsible for evoking the eosinophil leucocyte reaction present in the foreign normal cells was missing from the tumour cells.

Cells or tissues have been shown to contain antigens which determine the species, strain, tissue or organ specificity (Furth & Kabat, 1940, 1941 ; Triplett, 1962). Each of these consists of several components. It has also been shown that like normal cells, malignant cells contain all or almost all the normal species antigen (Moller, 1962) and also isoantigen (Klein, 1959) except in the case of very freely transplantable tumours (Amos, 1955). These antigenic components are known to play a vital role in the homograft reaction (Billingham *et al.*, 1954, 1956 ; Snell, 1957 ; Hellstrom, 1959 ; Brent *et al.*, 1961) and most foreign tumours are rejected by the host in a manner similar to the rejection of foreign normal tissue. However, there is much experimental evidence accumulating to show that many of the human and animal tumours lack organ or tissue specific antigen (Weiler, 1956 ; Vogt, 1958 ; Nairn *et al.*, 1960, 1962). Thus, considering the various experimental results described above, it is suggested that the local eosinophil leucocyte reaction to the foreign normal cells inoculation was due to the foreign tissue specific antigen (TSA) present in the cells and that the tumour cells, being devoid of TSA, failed to produce a similar reaction although the tumours were foreign to the host. This is supported by the fact that an intense local eosinophilia occurred following inoculation of normal liver submicrosomal fraction (fraction M) which is known to contain all or almost all of the tissue specific antigen (Vogt, 1958) but there were no similar reactions following inoculation of fractions prepared from the corresponding rat liver tumour.

Green (1954) in his immunological theory of carcinogenesis stressed that antigenic loss is an important feature of malignancy. This has been supported by the experimental evidence of many workers mentioned above. If the TSA of foreign normal tissue was responsible for inducing a local eosinophil leucocyte response then failure to produce a similar reaction by the tumour cells (native or foreign) gives further evidence to support the view that tumours lack TSA.

The fact that there was no local eosinophil leucocyte reaction to any of the pure suspensions of tumour cells, whether they grew or were rejected, indicates that the eosinophil response is not an essential participant in homograft rejection under these circumstances. However, the results of the study of the factors in the recipient mice affecting the local eosinophil response to foreign normal cells suggest that this response may be part of some other immune mechanism. The eosinophil response does not appear to be concerned with simple phagocytosis of the injected material, firstly because no signs of phagocytosis were observed among the eosinophils in this study, although ingested material was seen in macrophages and polymorphonuclear neutrophils, and secondly because no eosinophil leucocyte reaction was noted when the injected cells were of the same donor strain.

On the other hand, the difference between the intensity of eosinophilic response in untreated mice and mice presensitized by injecting cells from the donor strain is similar to the difference of antibody response to primary and secondary stimulation. The local eosinophil response was suppressed by cortisone and betamethazone, which also suppressed the immune response but not by phenergan, which suppressed anaphylactic evidence of the reaction of antigen with antibody. Newborn mice, which are immunologically immature and incapable of responding to antigenic stimulus by antibody production, did not show local eosinophilia. These facts considered together are consistent with the view that the local eosinophil leucocyte response may be part of some immune mechanism other than homograft rejection.

Thus from the above experimental results it was concluded that the eosinophil leucocytes are closely associated with the immunological phenomenon and are probably reacting in response to the tissue specific antigen which is foreign to the host. The possibility that native TSA might evoke a similar reaction if altered or modified in some way so as to act as foreign within the host is being investigated.

### SUMMARY

Local cellular reactions, with special reference to the eosinophil leucocytes, following subcutaneous inoculation with various isolated cells and cell fractions were studied. Intense local eosinophil leucocyte reaction to the genetically dissimilar normal cells was lacking in tumour cell inoculation. Factors such as age, presensitization or pretreatment of the recipient mice affect local eosinophil leucocyte reactions to the heterologous cells. The role of eosinophils in immune reactions is discussed.

I wish to thank Professor H. N. Green, Dr. J. O. Laws and Dr. H. M. Anthony for their helpful suggestions and valuable criticism. This work was partly supported by a grant from the Yorkshire Council of the British Empire Cancer Campaign.

### REFERENCES

ALLEN, H. C., WILLIAM, R. D., LOVINGOOD, C. C. AND ELLISON, E. H.—(1952) *Ann. Surg.*, **135**, 239.
AMOS, D. B.—(1955) *Ann. N.Y. Acad. Sci.*, **63**, 706.
ANDERSON, A. G.—(1953) *Science*, **117**, 627.
BARUA, B. D.—(1960) *Cancer Res.*, **20**, 1184.
BILLINGHAM, R. E., BRENT, L. AND MEDAWAR, P. B.—(1954) *Proc. Roy. Soc. B.*, **143**, 58. (1956) *Phil. Trans.* **239**, 357.
BLOOM, W. AND FAWCETT, D. W.—(1962) 'Textbook of Histology,' 8th Edition. Philadelphia, London (Sandaris Co. Ltd.).
BRANSTER, M. V. AND MORTON, R K.—(1957) *Nature, Lond.*, **180**, 1283.
BRENT, L., MEDAWAR, P. B. AND RUSZUIEWIEZ, V.—(1961) *Brit. J. exp. Path.*, **42**, 464.
BROWN, J. B. AND MCDOWELL, F.—(1942) *Ann. Surg.*, **115**, 1166.
CHUTNA, J.—(1957) *Transplant. Bull.*, **4**, 136.
DARCY, D. A.—(1952) *Phil. Trans.*, **276**, 463.
DEMPSTER, W. J.—(1953) *Brit. J. Surg.*, **40**, 447.
ESSELLIER, A. F., JEANNERET, R. L., CARMEN, L. AND WINKELSTEIN, N.—(1955) *Int. Arch. Allergy.*, **6**, 129.
FURTH, J. AND KABAT, E.—(1940) *Science*, **91**, 483.—(1941) *J. exp. Med.*, **74**, 247.

GREEN, H. N.—*Brit. med. J.*, ii, 1374.
HALBERG, F., HAMERSTON, O. AND BITTNER, J. J.—(1957) *Science*, **125**, 721.
*Idem*, VISSCHER, M. B. AND BITTNER, J. J.—(1953) *Amer. J. Physiol.*, **174**, 109.
HASKOVA, J., CHUTNA, J., HASEK, M. AND HART, J.—(1962) *Ann. N.Y. Acad. Sci.*, **99**, 602.
HELLSTROM, K. E.—(1959) *Transplant. Bull.*, **6**, 411.
HUMPHRIES, A. L. AND CAPTAIN, M. C.—(1956) *Ibid.*, **3**, 150.
KALTENBACH, J. P.—(1954) *Exp. Cell. Res.* **7**, 568.
KEILOVA, H. AND CHUTNA, J.—(1958) *Neoplasma*, **5**, 34.
KIDD, J. G.—(1950) *Proc. Inst. Med., Chicago*, **18**, 50.
KLEIN, E.—(1959) *Transplant. Bull.*, **6**, 420.
LOEB, L.—(1930) *Physiol. Rev.*, **10**, 547.
MEDAWAR, P. B.—(1945) *J. Anat.*, **79**, 157.—(1946) *Brit. J. exp. Path.*, **27**, 15.
MOLLER, E.—(1962) *Eighth Int. Cancer Congr. Abstr.* **95**.
MURGATROYD, L. B. AND GOSWAMI, P. K.—(1962) *J. med. Lab. Tech.*, **19**, 276.
MURPHY, J. B.—(1914) *J. Amer. med. Ass.* **62**, 145.
NAIRN, R. C., RICHMOND, H. G., MCENTERGERT, M. C. AND FOTHERGILL, J. H.—(1960) *Brit. med. J.*, ii, 1335.
*Idem*, FOTHERGILL, H. G., MCENTERGERT, M. C. AND RICHMOND, H. G.—(1962) *Ibid.*, i, 1791.
ROGERS, B. O.—(1954) *J. nat. Cancer Inst.*, **14**, 717.
*Idem*, CONVERSE, J. M., TAYLOR, A. C. AND CAMPBELL, R. M.—(1953) *Proc. Soc. exp. Biol., N.Y.*, **82**, 523.
SNELL, G. D.—(1957) *Annu. Rev. Microbiol.*, **11**, 489.
SPEIRS, R. S.—(1956) *Ann. N.Y. Acad. Sci.*, **73**, 283.
TITUS, J. L. AND SHORTER, R. G.—(1962) *Proc. Mayo Clin.*, **37**, 492.
TOOLAN, H. W. AND KIDD, J. G.—(1949) *Fed. Proc.* **8**, 373.
TRIPLETT, E. L.—(1962) *J. Immunol.*, **89**, 505.
VOGT, P.—(1958) *Nature, Lond.*, **182**, 1807.
WEILER, E.—(1956) *Brit. J. Cancer*, **10**, 553.